# An Improved Equivalent Simulation Model for CMOS Integrated Hall Plates

**DOI:** 10.3390/s110606284

**Published:** 2011-06-10

**Authors:** Yue Xu, Hong-Bin Pan

**Affiliations:** 1 School of Electronic Science & Engineering, Nanjing University, Nanjing 210093, China; 2 College of Electronic Science & Engineering, Nanjing University of Posts and Telecommunications, Nanjing 210003, China; E-Mail: yuex@njupt.edu.cn

**Keywords:** hall plate, simulation model, non-linear effects, Verilog-A

## Abstract

An improved equivalent simulation model for a CMOS-integrated Hall plate is described in this paper. Compared with existing models, this model covers voltage dependent non-linear effects, geometrical effects, temperature effects and packaging stress influences, and only includes a small number of physical and technological parameters. In addition, the structure of this model is relatively simple, consisting of a passive network with eight non-linear resistances, four current-controlled voltage sources and four parasitic capacitances. The model has been written in Verilog-A hardware description language and it performed successfully in a Cadence Spectre simulator. The model’s simulation results are in good agreement with the classic experimental results reported in the literature.

## 1. Introduction

Presently, CMOS integrated Hall magnetic sensors are widely used in many practical fields. Besides directly measuring the value of magnetic field, they are usually used to indirectly measure position, distance, speed, rotational angle or an electric current [1,2]. For instance, they can act as an automotive vehicle speed sensor, a replacement for mechanical switches, a brushless control for DC motors, and so on. Unfortunately, CMOS integrated Hall sensors have traditionally suffered from a lot of non-idealities, such as low sensitivity, large offset, temperature drifts, non-linearity and packaging stress influence *etc.*, which severely deteriorates their performance [3]. As a consequence, CMOS integrated Hall devices must depend on the processing circuit for offset and noise cancellation, temperature compensation and non-linearity correction. In order to facilitate the simulation analysis of electrical circuit with integrated Hall devices, it is necessary to extract a precise simulation model to take into account important physical effects and technological influences. Furthermore, the extracted model should be simple and conveniently implemented in standard SPICE-like EDA tools.

Several compact simulation models of Hall elements have been put forward. Previously reported 4-resistance Wheatstone bridge models don’t fully take into account correlative physical and geometrical effects such as non-linear conductivity, junction effect, temperature drift, frequency-response, noise behavior and device shape-dependent sensitivity [4,5]. Later, Dimitropoulos *et al.* proposed a completely scalable lumped-circuit model to analyze all those effects, except for the influence of packaging stress [6]. The basic component for the lumped-circuit model consists of JFETs and current-controlled current sources. The number of these components can be freely increased to achieve the required accuracy at the expense of computation efficiency. However, this macro model needs an accurate JEFTs device model which normally cannot be provided by the standard CMOS technology. Recently, Madec *et al.* developed a compact model of a cross-shaped horizontal integrated Hall sensor [7]. It uses six sub-components to accurately model the non-linear resistance, allowing for the influence of space charge region modulation due to sensor bias. Unfortunately, it cannot consider sensitivity drifts, temperature drifts and influence of mechanical stress on offset. Besides, the resistance computation of the model has to be fed by empirical parameters through FEM (finite element method) simulation.

In this paper, an accurate 8-resistance simulation model for a cross-shaped CMOS-integrated Hall plate is developed. To be conveniently used by circuit designers, this model is improved by replacing the JFETs with passive non-linear resistances and depletion capacitances. It takes into account voltage dependent non-linear effects, geometrical effects, temperature effects, and packaging stress influence, *etc.* Since we mainly deal with the magnetic sensors operating in a weak magnetic field in this work, two additional strong magnetic field related effects, namely magneto-resistance and carriers scattering, are not included in this model. The model has been written in Verilog-A hardware description language and was successfully tested in a Cadence Spectre simulator. This paper is organized as follows: in Section 2, we introduce the structure of the model and analyze the important physical effects of the Hall device with the basic equations. Furthermore, the detailed computation of device parameters in this model is presented. In Section 3, the simulation results of the model are compared with the classic experimental results reported in the literature. Section 4 summarizes this paper with some ideas for future work.

## 2. The Improved Compact Model

**Figure 1.**
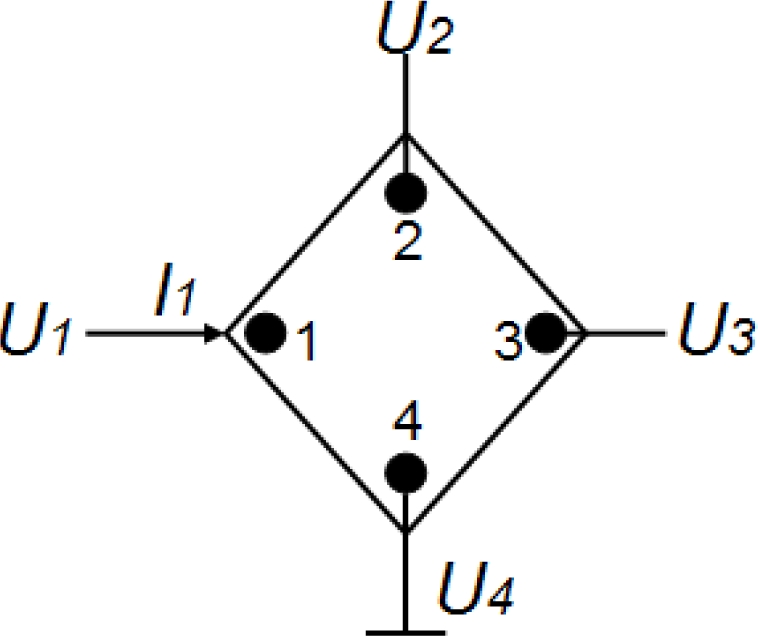
Diagram of measuring Z-matrix for a 90° symmetry Hall plate.

To be compatible with the spinning current techniques for reducing Hall offset [3], 90° symmetry Hall plates with square or cross-shaped structures are usually recommended. We can obtain the Z-matrix for the 90° symmetry Hall plates with four contacts illustrated in Figure 1 as follows [8]:

(1) $$Z=\left(\begin{array}{lll} Z_{11} & Z_{12} & Z_{13}\\ Z_{12} & 2Z_{12} & Z_{12}\\ Z_{13} & Z_{12} & Z_{11} \end{array}\right)$$

**Figure 2.**
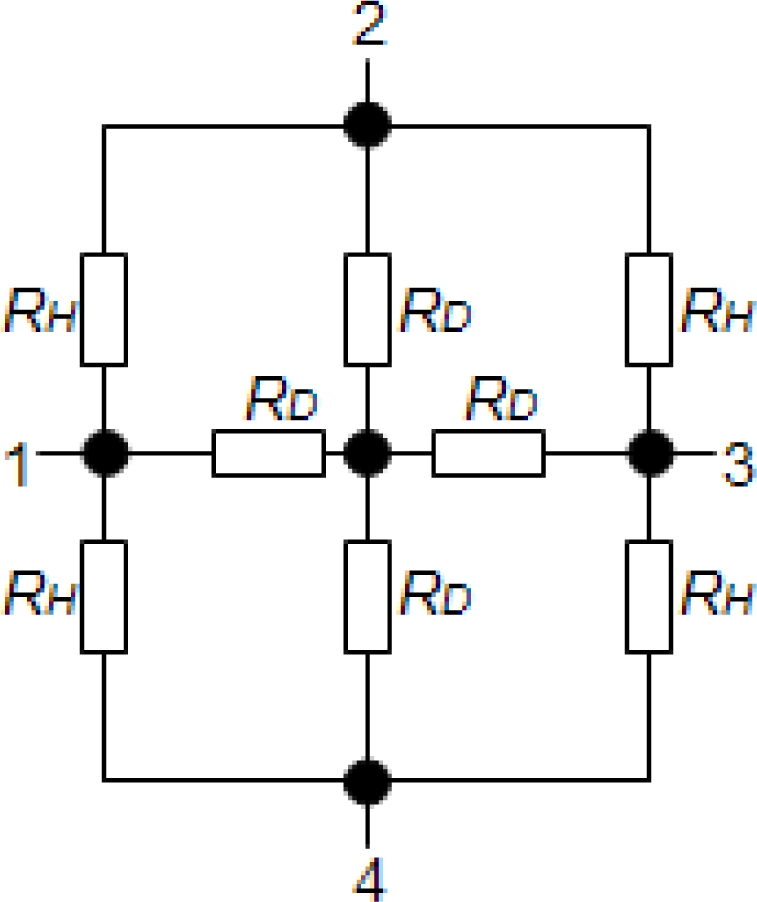
An equivalent model topology for the 90° symmetry Hall plate.

If the fourth contact is applied to the reference ground, the Z-matrix of the 90° symmetry Hall plate is only decided by three parameters *Z*_11_, *Z*_12_, *Z*_13_. If the input current *I*_1_ is applied to the first contact, the three measuring potentials shown in Figure 1 have the following relation: *U*_1_ – *U*_2_ = *U*_3_, and then we can obtain *Z*_11_ – *Z*_12_ = *Z*_13_. As a result, 90° symmetry Hall plates require at least two types of resistances to model their electrical properties. Thus, an 8-resistance model topology for the 90° symmetry Hall plate is suggested, which is illustrated in Figure 2. Its Z-matrix at zero-magnetic field is expressed by:

(2) $$Z=\frac{1}{8}\frac{2R_{H}R_{D}}{2R_{D}+R_{H}}\left(\begin{array}{ccc} 6+R_{H}/R_{D} & 4 & 2+R_{H}/R_{D}\\ 4 & 8 & 4\\ 2+R_{H}/R_{D} & 4 & 6+R_{H}/R_{D} \end{array}\right)$$

However, in the conventional 4-resistance Wheatstone bridge model [4,5], the diagonal resistances *R_D_* are often neglected. The value of the resistance between two adjacent contacts is not accurate because current lines linking the two adjacent contacts do not flow across the center of the device. But this problem can be solved well in the 8-resistance model so that the simulation accuracy can be improved.

### 2.1. The Structure of the Model

**Figure 3.**
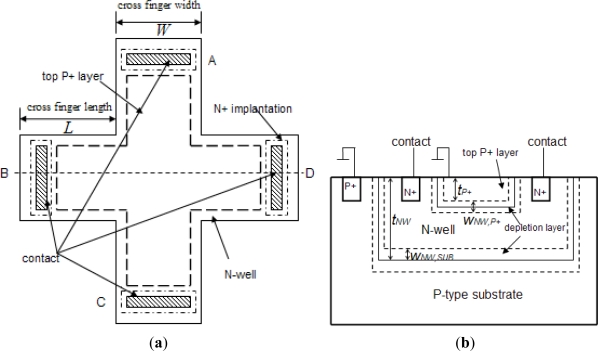
Cross-shaped Hall plate fabricated in standard CMOS technology. (**a**) Top view; (**b**) View with the cross-section along B and D contacts.

**Figure 4.**
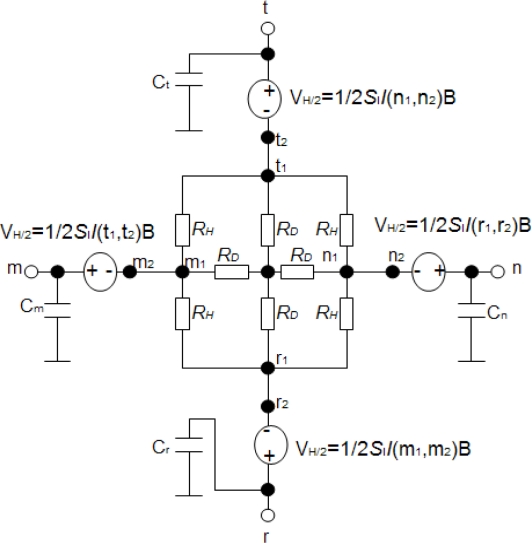
A simplified model for the CMOS integrated cross-shaped Hall plate.

The 90° symmetry cross-shaped Hall plate (see Figure 3) is most widely used because of its high sensitivity and immunity to alignment tolerances resulting from the fabrication process. Its fabrication technology is fully compatible with the standard CMOS process. As shown in Figure 3(a), the active area of the cross-shaped Hall plate is usually realized by a weakly doped N-well diffusion region. The N-well is isolated from the P-type substrate by the reverse-biased well/substrate p-n junction. A shallow heavily doped top P+ layer often covers the surface of active area to decrease the flicker noise and the surface losses. It is normally formed to create the source and drain regions of PMOS transistors [9]. In addition, four contact regions are highly N+ doped to reduce the contact resistances in the source and drain formation processing step for NMOS transistors. Based on the basic model topology shown in Figure 2, a new simplified 8-resistance model for the CMOS integrated cross-shaped Hall plate is developed, as shown in Figure 4. Compared with Dimitropoulos’ model, the simulation model is improved by replacing the JFETs by N-well body resistances and depletion capacitances. The four depletion capacitances are added into the model to simulate the transient behavior of the Hall plate. Besides, there are four controlled voltage sources *V*_*H*/2_ to model the Hall voltage. Each Hall voltage source *V*_*H*/2_ is controlled by the electrical current flowing through the nearer contact.

In order to determine the resistance values of *R_H_* and *R_D_* in our model, a new and simple computation method is proposed in contrast to the FEM simulation method [7]. It is well known that it is best to measure the N-well sheet resistance *R_s_* according to the Van-der-Pauw method. Although the Van-der-Pauw method requires that the contacts of Hall device be point-like, it has been reported that cross-shaped Hall plate with a finger length to width ratio larger than 1:1 can give an accurate *R_s_* value with an error of less than 0.1% [10]. Usually, the required ratio of finger length to width can be fulfilled for achieving a high current related sensitivity. Thus, in the case of symmetric Hall plates, the sheet resistance can be determined by measuring the resistance value *R_AB,CD_* in term of Van-der-Pauw method [11]:

(3) $$R_{AB,CD}=\frac{\text{ln}2}{\pi }R_{s}$$

where *R_AB,CD_* = *V_CD_* / *I_AB_* presents the voltage difference between contacts C and D dividing the current flowing from contact A to contact B. The contacts of A, B, C and D are shown in Figure 3(a).

On the other hand, according to the structure of the model illustrated in Figure 4, the resistance *R_AB,CD_* is calculated by:

(4) $$R_{AB,CD}=\frac{R_{H}}{4}\frac{2R_{D}-R_{H}}{2R_{D}+R_{H}}$$

The internal resistance between two diagonal contacts is given by:

(5) $$\frac{2R_{D}R_{H}}{2R_{D}+R_{H}}=(2\frac{L}{W}+\frac{2}{3})R_{s}$$

Here, (2*L/W*+2/3) is the effective square number of the N-well resistance. *L* and *W* are the finger length and finger width of cross-shaped Hall plate, respectively. The center square number is approximately reduced to 2/3 as the two fingers for sensing Hall signal are placed in parallel. Considering the Equations (3), (4) and (5), finally we can obtain:

(6) $$R_{H}=\frac{2R_{s}}{\pi }\left[\left(2\frac{L}{W}+\frac{2}{3}\right)\pi -2\text{ln}2\right]$$

(7) $$\frac{R_{H}}{R_{D}}=2-\frac{8}{\pi }\frac{\text{ln}2}{2L/W+2/3}$$

The N-well sheet resistance *R_s_* is calculated by:

(8) $$R_{s}=\frac{1}{q\mu_{n}N_{D,NW}t_{eff}}$$

Here, *N_D,NW_* is the N-well doping concentration, *t_eff_* is the effective depth of Hall plate. As shown in Figure 3(b), it is equal to:

(9) $$t_{eff}=t_{NW}-t_{P+}-w_{NW,SUB}-w_{NW,P+}$$

where *t_NW_* is the depth of N-well implantation region, *t_P+_* is the thickness of the top P+ layer, *w_NW,SUB_* is the bottom depletion region situated in between N-well and P-type substrate, *w_NW,P+_* is the upper depletion region situated in between N-well and top P+ layer.

Note that there are two main parasitic capacitances distributed across the Hall device body: (1) the reverse-biased upper depletion capacitance between the top P+ layer and N-well; (2) the reverse-biased bottom depletion capacitance between the N-well and p-type substrate. Usually the top P+ layer and P-type substrate are applied to ground together, thus they are connected in parallel physically. Unfortunately, the parasitic capacitances may limit the switching frequency for the spinning current offset reduction method. In order to simulate the complete ac behavior of the Hall plate, these parasitic depletion capacitances should be included in the model. Assuming the one-sided abrupt junctions, each depletion capacitance per unit area is calculated by following Equation (5):

(10) $$C_{pn}=\sqrt{\frac{q\varepsilon_{si}\cdot N_{D,NW}N_{A}}{2(N_{D,NW}+N_{A})}}{\left[V_{bi}-U_{pn}-\frac{2KT}{q}\right]}^{-1/2}$$

Here, *V_bi_* is the built in potential of PN junction, *N_D,NW_* is the doping of N-well, and *N_A_* is the doping of top P+ layer or P-type substrate.

### 2.2. Hall Voltage and Magnetic Sensitivities

When a magnetic field *B* is orthogonally applied on a device plane and two diagonal contacts are biased with a current *I*, the Hall effect takes place. Then the hall voltage *V_H_* appears on two additional contacts, it is equal to:

(11) $$V_{H}=S_{I}IB$$

where *S_I_* is the current related sensitivity. It depends on device geometry (geometrical correction factor G and Hall plate effective thick *t_eff_*) and technology parameters (N-well doping *N_D,NW_* and Hall *r_H_*) [12]. It is defined as:

(12) $$S_{I}\frac{Gr_{H}}{qN_{D,NW}t_{eff}}$$

When Hall plate is biased with a voltage source *V*, the Hall voltage is expressed by voltage related sensitivity *S_V_*:

(13) $$V_{H}=S_{V}VB$$

where *S_V_* = *μ_H_G* / *N_square_* · *μ_H_* = *r_H_μ_n_*, it is the Hall mobility, *μ_n_* is the electron mobility, and *N_square_* is the equivalent square number of N-well diffusion resistance between two diagonal contacts.

The impact of the Hall devices geometry on Hall voltage is modeled by a geometrical correction factor. For a cross-shaped Hall plate, it can be calculated by using a conformal mapping [12]:

(14) $$G=1-5.0267\frac{\theta_{H}}{\text{tan}(\theta_{H})}e^{-\frac{\pi }{2}\frac{W+2L}{W}}$$

where *θ_n_* = tan^−1^(*μ_H_B*), it is defined as the Hall angle. If *W* / 2*L* ≤ 0.39, *G* has an accuracy better than 0.5% [12].

In our model illustrated in Figure 4, each Hall voltage *V*_*H*/2_ is modeled by using the current-controlled voltage sources with the following equation:

(15) $$V_{H/2}=\frac{1}{2}S_{I}I(n_{1},n_{2})B$$

with *I*(*n*_1_, *n*_2_) being current flowing between the contacts *n*_1_ and *n*_2_.

### 2.3. Voltage Dependent Non-Linear Effect

It is well known that the thickness of depletion region is obviously changed by the reverse biased PN junction. Therefore, both sheet resistance and magnetic sensitivity suffer from a strong voltage non-linearity dependence. Since the doping concentration of the P+ top layer is obviously higher than that of the P-type substrate, the thickness variation of the upper depletion region modulated by reverse biased voltage can be approximately ignored. Using Equation (8), which is extended by the voltage dependent *t_eff_*, and a Taylor expansion results up to second order are given by [5]:

(16) $$\begin{array}{l} R_{s}(U_{pn})=\frac{1}{q\mu_{n}N_{D,NW}(t_{eff}^{*}-\sqrt{k_{1}V_{bi}})}+\frac{1}{2}\frac{\sqrt{k_{1}V_{bi}}}{q\mu_{n}N_{D,NW}{\left(t_{eff}^{*}-\sqrt{k_{1}V_{bi}}\right)}^{2}V_{bi}}U_{pn}\\ +\frac{-\frac{1}{8}\frac{\sqrt{k_{1}V_{bi}}}{(t_{eff}^{*}-\sqrt{k_{1}V_{bi}})V_{bi}^{2}}+\frac{1}{4}\frac{k_{1}}{{\left(t_{eff}^{*}-\sqrt{k_{1}V_{bi}}\right)}^{2}V_{bi}}}{q\mu_{n}N_{D,NW}\left(t_{eff}^{*}-\sqrt{k_{1}V_{bi}}\right)}U_{pn}^{2}\\ =R_{s}(0V)\cdot (1+BBR_{1}\cdot U_{pn}+BBR_{2}\cdot U_{pn}^{2}) \end{array}$$

where *R_s_*(0*V*) is the zero-biased N-well sheet resistance, *BBR*_1_ and *BBR*_2_ are the first and second voltage dependency of resistance coefficients, respectively. $k_{1}=\frac{2{ɛ}_{si}}{q}\cdot \frac{N_{A,SUB}}{(N_{A,SUB}+N_{D,NW})N_{D,NW}}$, and $t_{eff}^{*}=t_{NW}-t_{P+}-w_{N,P+}(0V)$. Here, *w*_*N,P*+_ (0*V*) is the upper depletion region with zero-bias.

With the same calculation method, the current related sensitivity is modeled by:

(17) $$S_{I}(U_{pn})=S_{I}(1+BBS_{1}\cdot U_{pn}+BBS_{2}\cdot U_{pn}^{2})$$

where *BBS*_1_ and *BBS*_2_ denote the first and second voltage dependent coefficients of sensitivity, respectively.

### 2.4. Temperature Effect

We know that the temperature drift has serious effects on the equivalent N-well resistance, sensitivities and offset of the Hall device. The temperature behavior of N-well sheet resistance can be well approximated by the second order polynomial:

(18) $$R_{s}(U_{pn},T)=R_{s}(U_{pn},300K)\cdot [1+R_{TC1}\cdot (T-300K)+R_{TC\;2}\cdot {\left(T-300K\right)}^{2}]$$

where *R*_*TC*1_ and *R*_*TC*2_ are temperature coefficients of N-well resistance. These parameters can be directly obtained from foundry technological files. *R_S_(U_pn_,300K)* is the sheet resistance at the room temperature.

Since the thermal expansion of silicon is merely 2.6 ppm/°K and the G and *t_eff_* are considered as temperature independent, the thermal drift efficient of current related sensitivity can be given by [12,13]:

(19) $$\alpha_{SI}=\frac{1}{S_{I}}\frac{dS_{I}}{dT}=\alpha_{rH}-\alpha_{N}$$

where, *α_rH_* and *α_N_* are the temperature coefficient of the Hall factor and the carrier concentration of N-well, respectively. For a N-well doping of 4 × 10^16^ cm^−3^, *α_rH_* increases from 0 to 500 ppm/°K in a industrial temperature range (240 K–400 K) [12]. With a lower doping of N-well, *α_rH_* is almost independent of N-well doping and the temperature, showing an approximately 700 ppm/°K constant value [12]. On the contrary, *α_N_* decreases from 500 ppm/°K to 50 ppm/°K for the N-well doping of 4 × 10^16^ cm^−3^ in the same temperature range [13]. As a result, *α_N_* could just right compensate *α_rH_* at the room temperature, and zero temperature coefficient of sensitivity could be obtained. Considering the temperature dependency of *α_rH_* and *α_N_*, the drift of the current related sensitivity is about in the range of ±500 ppm/°K throughout the industrial temperature range. For the other doping values of N-well, *α_rH_* and *α_N_* can be obtained by referring to relevant data. Thus, considering this thermal drift effect of the sensitivity, Equation (20) can be rewritten as:

(20) $$S_{I}(T,U_{pn})=S_{I}(U_{pn})[1+\alpha_{SI}\cdot (T-300K)]$$

### 2.4. Piezo-Resistance and Piezo-Hall Effects

When a Hall plate is assembled, its performance is deteriorated by two physical stress-related effects, *i.e.*, piezo-resistance and piezo-Hall effects. The piezo-resistance effect due to packaging stress provokes relative variations of N-well resistances. In combination with technology variation, junction field effects and temperature drift, it is the main source of offset. The sensitivity is also impacted by packaging stress. The variation of sensitivity is called piezo-Hall effect. Considering this effect, Equation (20) can be rewritten as [14,15]:

(21) $$S_{I}(\sigma ,T,U_{pn})=S_{I}(T,U_{pn})[1+P_{12}\cdot (\sigma_{x}+\sigma_{y})]$$

where *σ_x_* and *σ_y_* are the mechanical stress in the plane parallel to the Hall plate surface, *P*_12_ denotes the piezo-Hall coefficient tensors in x-y plane, which is estimated at 40 × 10^−11^ Pa^−1^ for the N-well (4 × 10^16^ cm^−3^) [12].

Since the mechanical stress changes with temperature, the temperature coefficient of sensitivity illustrated in Equation (19) can be rewritten as:

(22) $$\alpha_{SI}=\alpha_{rH}-\alpha_{N}-\alpha_{piezo-Hall}$$

For a plastic packaging, the temperature coefficient related to piezo-Hall effect can be defined by [13]:

(23) $$\alpha_{piezo-Hall}=-C_{1}\cdot P_{12}\cdot E_{pg}\cdot (\alpha_{pg}-\alpha_{silicon})$$

where *E_pg_* is the modulus of elasticity of molding compound, *α_pg_* and *α_silicon_* is the thermal expansion coefficients of molding-compound and silicon, *C*_1_ is a designed-dependent geometric constant. For a typical plastic package such as TSSOP, we can get *C*_1_ ≈ 6 [13], so the approximate *α_piezo–Hall_* value of −450 ppm/°K can be estimated [12].

## 3. Comparing Results of Simulation with Experimental Results

The new simulation model code has been written in behavioral Verilog-A language and was tested on a Cadence Spectre simulator tool using AMS 0.8 μm CMOS technological parameters (shown in Table 1) [12]. The finger width and finger length of the cross-shaped Hall plate are designed to 40 μm and 60 μm, respectively.

**Figure 5.**
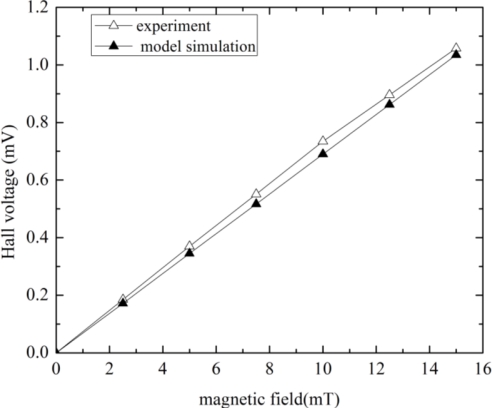
Comparisons between the measurements and the model simulation for the output Hall voltage with 1 mA biasing.

To show the correctness and accuracy of this model, the corresponding experimental results of the Hall plate fabricated using the same technology given in the literature [12] are compared with the model’s simulation results. First, we performed the simulation of Hall voltage *versus* magnetic field at room temperature. The simulated and test results when the input bias current is 1 mA and the Hall plate is liberated of packaging stress, are plotted in Figure 5. It can be observed that the simulated Hall voltage is proportional to magnetic field intensity. When the magnetic field intensity is 2.5, 5, 7.5, 10, 12.5 and 15 mT, the simulated Hall signal is 0.172, 0.345, 0.517, 0.69, 0.862 and 1.035 mV, respectively. While the value of the measured Hall voltage is 0.185, 0.37, 0.551, 0.735, 0.896 and 1.058 mV for the corresponding magnetic field intensity. We can see that the simulated current-related sensitivity is 69 V/AT, while the tested result is 75 V/AT. A very good agreement is thus obvious in Figure 5. If a mechanical stress in the CMOS Hall plate is estimated at σ_x_ = σ_y_= −70 MPa for a typical plastic packaging [14], which will lead to a variation of the simulated magnetic sensitivity of about 5% at room temperature compared with the stress-free sensitivity. Secondly, the simulation of the N-well sheet resistance *versus* input voltage was implemented at room temperature without packaging stress influence.

**Figure 6.**
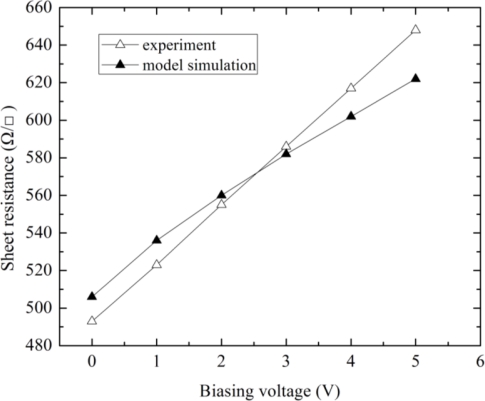
Comparisons between the measurements and the model simulation for the sheet resistance of N-well dependence of input voltage.

Figure 6 illustrates the comparison between the simulated and tested sheet resistances per square *versus* variation of input voltage (sweeps from 0 V to 5 V). The measured sheet resistance per square changes from 493 Ω/γ to 648 Ω/γ. By comparison, the simulated sheet resistance per square changes in the range (506 Ω/γ–622 Ω/γ) with a small error. In addition, the characteristics of magnetic sensitivity *vs.* temperature drift were simulated.

**Figure 7.**
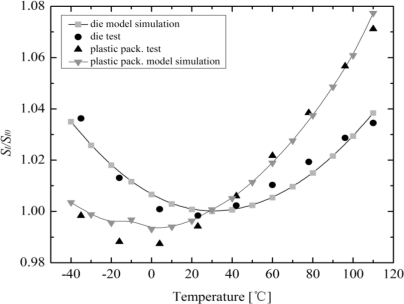
Comparisons between the measurements and the model simulation for the relative variation of the current-related sensitivity as a function of temperature.

The measured and simulated relative variations of the current-related sensitivity related to the value at room temperature as a function of temperature for the zero-stress mounting of the Hall plate is demonstrated in Figure 7. In this temperature dependence of sensitivity simulation, we assume the zero temperature coefficient *α_SI_* of Hall plate takes place at 27 °C, and *α_SI_* linearly changes from −500 ppm/°K to +500 ppm/°K in the temperature range from −40 °C to 110 °C. It is obvious that a good accordance is achieved between simulation and experimental results for a die absence of packaging stress influence. Meanwhile, both simulated and tested results of the thermal drift of *S_I_* influenced by the plastic packaging stress as a function of temperature are also shown in Figure 7. The simulated thermal drift of *S_I_* for a typical plastic package (TSSOP) is also in good agreement with the measured results. It should be pointed out that the piezo-resistance and piezo-Hall effects in packaged Hall sensors are very complex issues, which cannot accurately be modeled by only a small number of key physical and technological parameters. Therefore, a very accurate simulation result cannot be achieved in some cases.

**Figure 8.**
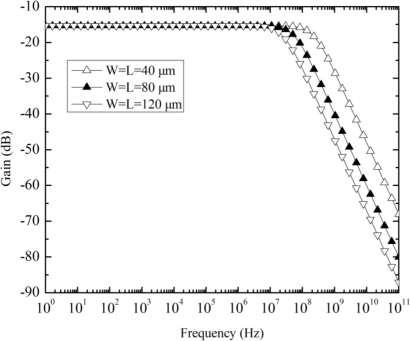
Ac simulation gain *versus* frequency for three Hall plates with different dimensions.

Finally, the ac simulation of the Hall plate was performed at 3 V DC bias. The ratio of finger length to finger width is fixed to 1, while the finger length is taken as a parameter, changing from 40 μm to 120 μm with a step of 40 μm. The simulation results in Figure 8 show that the smaller Hall plate has the higher corner frequency and the −3 dB bandwidth highly exceeds the one MHz range for the largest Hall plate, indicating that any limited frequency response of Hall plate within the working range (usually below 1 MHz) cannot be observed.

## 4. Conclusions

An equivalent circuit simulation model for a CMOS-integrated Hall plate has been improved. The structure of the model consists of a passive network, including eight non-linear resistances, four depletion capacitances and four current-controlled voltage sources. The model completely takes into account the non-linear conductivity effects, geometrical effects and temperature effects. Meanwhile, the packaging stress influence on Hall plates is also considered to a certain degree. In addition, the model only needs a small number of key physical and technological parameters. The model has been implemented in Verilog-A hardware description language and was successfully tested with the standard EDA tool Cadence. For testing the model correctness and accuracy, the model simulation of a Hall plate were performed using AMS 0.8 μm CMOS technology parameters and are compared with the measured results reported in the literature [12]. A very good agreement is obtained. It should be noted that if those key technological parameters such as N-well sheet resistance, Hall mobility, *etc.* can be calibrated by the measurements of the Hall sensors fabricated in a standard CMOS technology line, more accurate model simulation results could be achieved.

## Figures and Tables

**Table 1.** Model parameters (using AMS 0.8 μm CMOS process) [12].

Parameters Definition Default value
*N_D,NW_* Doping in N-well 4 × 10^16^ cm^−3^
*N_A,P+_* Doping in top P+ layer 1 × 10^20^cm^−3^
*N_A,SUB_* Doping in substrate 1 × 10^16^cm^−3^
*t_NW_* Depth of N-well 4 μm
*t_P+_* Depth of top P+ layer 0.3 μm
*μ_n_* Electrons mobility 950 cm^2^/V.s
*μ_H_* Hall mobility 1,200 cm^2^/V.s
*R_TC1_* First temperature coefficient of N-well resistance 1%/°K
*R_TC1_* Second temperature coefficient of N-well resistance 20 ppm /°K
*P_12_* Piezo-Hall coefficient 40 × 10^−11^ Pa^−1^
*α_SI_* Temperature coefficient of *S_I_* ±500 ppm/°K
*α_piezo-Hall_* Temperature coefficient of piezo-Hall effect −450 ppm/°K
